# Academic- and Family-Related Correlates of Anxiety Among College-Going Adolescents in Central India: A Cross-Sectional Study

**DOI:** 10.7759/cureus.114386

**Published:** 2026-08-11

**Authors:** Heena Chawla, Umesh Sinha, Rajkumar Agriya, Hitesh Ranasaria

**Affiliations:** 1 Community Medicine, Dr. Laxminarayan Pandey Government Medical College, Ratlam, IND; 2 Orthopedics, Virendra Kumar Sakhlecha Government Medical College, Neemuch, IND

**Keywords:** academic factors, adolescent anxiety, college students, cross-sectional study, dass-21, family factors, mental health

## Abstract

Background: Anxiety is the most common mental health disorder among adolescents worldwide. In India, intense academic competition and evolving family dynamics add to this burden, yet systematic data from smaller central Indian districts are scarce.

Objective: This study aims to determine the prevalence and severity of anxiety among college-going adolescents aged 17-19 years in Ratlam district, Madhya Pradesh, and to examine its association with academic- and family-related factors.

Methods: In this cross-sectional observational study, 550 first-year students were enrolled from 10 colleges (five professional, five nonprofessional) through multistage random sampling between June 2023 and November 2024. Depression, anxiety, and stress were measured with the validated Depression Anxiety Stress Scale-21 (DASS-21). Family-related factors included family type, parental marital status, sibling count, and paternal education, while academic-related factors included college type. Associations were assessed with the chi-square test and Cramér's V, and factors independently associated with clinically significant anxiety were assessed using binary logistic regression, yielding adjusted odds ratios (AORs) with 95% confidence intervals (CIs), using R (version 4.3); p < 0.05 was considered significant.

Results: Clinically significant anxiety was recorded in 178 participants (32.36%) (mild: 110 (20.00%); moderate: 57 (10.36%); severe: 11 (2.00%)), making anxiety the most prevalent DASS-21 domain ahead of stress (116; 21.09%) and depression (62; 11.28%). On multivariable analysis, female sex (AOR: 2.26; 95% CI: 1.45-3.52; p < 0.001), professional-college enrolment (AOR: 1.45; 95% CI: 1.05-2.01; p = 0.024), and hostel residence (AOR: 1.68; 95% CI: 1.15-2.45; p = 0.007) were independently associated with higher odds of clinically significant anxiety, whereas joint-family structure was associated with lower odds (AOR: 0.62; 95% CI: 0.42-0.91; p = 0.015). Nuclear-family students showed the highest anxiety prevalence (113 of 301; 37.54%) compared with joint-family students (59 of 230; 25.70%). Music (226; 41.09%) and social connection (95; 17.27%) were the most frequently used coping strategies, whereas physical exercise was rarely used (8; 1.45%).

Conclusions: Academic- and family-related factors were associated with anxiety among college-going adolescents. These findings support the need for campus-based mental-health screening, accessible counselling services, and promotion of healthy coping strategies, including physical activity.

## Introduction

Anxiety is characterized by excessive fear, worry, and apprehension that are disproportionate to the actual situation and sufficiently persistent to interfere with daily functioning. Common symptoms include excessive worrying, restlessness, difficulty concentrating, irritability, muscle tension, sleep disturbances, and autonomic manifestations such as palpitations and sweating. According to the Diagnostic and Statistical Manual of Mental Disorders, Fifth Edition, Text Revision (DSM-5-TR), the diagnosis of an anxiety disorder requires characteristic symptoms that persist for the specified duration, cause clinically significant distress or impairment in social, occupational, or other important areas of functioning, and cannot be better explained by another medical or psychiatric condition [1].

According to the World Health Organization (WHO), roughly one in seven individuals aged 10-19 years lives with a diagnosable mental health condition, and anxiety is the single most frequently identified disorder in this group, affecting an estimated 4.1% of those aged 10-14 years and 5.3% of those aged 15-19 years [2]. Anxiety disorders thus rank among the foremost contributors to adolescent morbidity worldwide.

In India, a recent systematic review and meta-analysis reported that the pooled prevalence of anxiety disorders among adolescents ranged from 23% to 29% in studies with low risk of bias, indicating a substantial burden of anxiety in this age group [3]. As adolescence is a period of transition, young people are especially vulnerable to mental health disturbances. The convergence of pubertal changes, evolving peer and social ties, identity development, and escalating academic pressure can precipitate or worsen anxiety during these years. Within India, this burden is further intensified by a highly competitive examination environment, career-related pressure, high parental expectations, and socioeconomic disparities [4].

Among the factors associated with anxiety in adolescents, academic- and family-related characteristics have received considerable attention. Academic stress, encompassing examination pressure, heavy course loads, fear of failure, and career uncertainty, has been repeatedly linked to anxiety among Indian college students, with reported prevalence figures varying widely across settings and screening tools [5,6]. Family environment encompasses family structure (nuclear, joint, or extended), parental marital stability, parental education, and the overall quality of intrafamilial relationships. Emotional warmth, consistent support, and clear communication act as protective factors against anxiety, whereas parental conflict, separation, punitive parenting, and adverse socioeconomic circumstances heighten psychological vulnerability [7,8].

Systematic data from central India, particularly from smaller districts such as Ratlam in Madhya Pradesh, are largely absent from the existing literature. Given Ratlam's mixed urban-rural demographic profile and its expanding higher-education sector, it represents a meaningful and understudied population. This study was designed to quantify the prevalence and severity of anxiety among college-going adolescents aged 17-19 years in Ratlam district and to examine its association with selected academic, family, and sociodemographic factors.

The primary objective of this study was to determine the prevalence and severity of anxiety among college-going adolescents aged 17-19 years in Ratlam district, Madhya Pradesh. A secondary objective was to examine the association of anxiety with selected academic-, family-related, and sociodemographic factors, including college type, family structure, parental characteristics, residence, and socioeconomic status. By identifying factors associated with anxiety in this understudied population, the study aims to provide evidence that can guide institution-based mental health screening, counselling services, and preventive interventions for college students.

## Materials and methods

Study design and setting

This was a cross-sectional observational study conducted in colleges within Ratlam district, Madhya Pradesh (coordinates: 23.33°N, 75.05°E; area: 4,861 km²; 2011 census population: 1,455,069). Data were gathered over 18 months, from June 2023 to November 2024. Ethical clearance was obtained from the Institutional Ethics Committee of Government Medical College, Ratlam (approval no. GMC/RATLAM/2023/IEC/Approval/08, dated 15/06/2023) prior to commencement.

Study population, sample size, and sampling strategy

The required sample size for estimating a single proportion was derived from the formula n = Z²P(1-P)/d², in which Z = 1.96 corresponded to 95% confidence, P denoted the expected anxiety prevalence of 14.5% reported by Mishra et al. [4], and d was the absolute precision of 3%. This yielded a minimum requirement of 529 participants. To ensure adequate representation across the selected colleges and account for possible nonresponse, 550 students were targeted, with 55 students selected from each college [9].

Multistage random sampling was used. In stage 1, 10 colleges were selected by simple random sampling from the district roster, comprising five professional and five nonprofessional colleges. In stage 2, a list of all eligible first-year students was obtained from each selected college. The sampling interval (k) was calculated by dividing the total number of eligible students by the required sample size (55). A random starting point between 1 and k was selected using a random number table, after which every kth student on the list was recruited until the required sample size was achieved.

Inclusion and Exclusion Criteria for Colleges

Eligible colleges were undergraduate colleges located within Ratlam district offering regular first-year academic programs and providing institutional permission for participation. Both professional and nonprofessional colleges were eligible for inclusion. Colleges that declined institutional permission or did not have eligible first-year students were excluded.

Inclusion and Exclusion Criteria for Students

Inclusion criteria: The inclusion criteria included first-year undergraduate students aged 17-19 years, enrolled in one of the selected colleges, and provided informed consent (and parental consent/assent where applicable).

Exclusion criteria: The exclusion criteria included students absent on the day of data collection and students with incomplete questionnaires (if this occurred).

Study Instruments

Data were collected face-to-face using a standardized, prevalidated, semistructured questionnaire made up of two parts. The first part was a sociodemographic proforma that recorded age, sex, religion, place of residence (hostel or nonhostel), type of college, caste category, parental education and occupation, family type, number of siblings, and parental marital status; socioeconomic position was graded with the Modified Kuppuswamy socioeconomic scale updated for 2024 [10].

The second part comprised the 21-item Depression Anxiety Stress Scale (DASS-21), a validated self-report instrument derived from the original 42-item DASS and widely used to assess symptoms of depression, anxiety, and stress in clinical and community populations [11,12]. The instrument contains 21 items, with seven items each assessing depression (items 3, 5, 10, 13, 16, 17, and 21), anxiety (items 2, 4, 7, 9, 15, 19, and 20), and stress (items 1, 6, 8, 11, 12, 14, and 18). Each item is rated on a four-point Likert scale ranging from 0 ("Did not apply to me at all") to 3 ("Applied to me very much or most of the time"), reflecting symptoms experienced during the previous week. The DASS-21 has demonstrated excellent psychometric properties, with reported Cronbach's alpha values ranging from 0.88 to 0.94 for the total scale and approximately 0.82-0.85 for the anxiety subscale, supporting its reliability and validity across diverse populations [12,13]. For descriptive purposes, raw anxiety scores were graded as normal (0-3), mild (4-5), moderate (6-7), severe (8-9), or extremely severe (≥10), with a score of ≥4 considered indicative of clinically significant anxiety. Raw anxiety scores were analyzed without multiplication because raw-score cutoffs equivalent to the standard DASS-21 thresholds have previously been reported [12].

Statistical analysis

Data were compiled in MS Excel (Microsoft Corporation, Redmond, Washington, United States) and analyzed with R (version 4.3). Categorical variables were expressed as frequencies and percentages. The chi-square test was used to examine associations between categorical variables and anxiety severity, and the strength of each association was quantified with Cramér's V. A binary logistic regression model was then fitted with clinically significant anxiety (yes/no) as the outcome. Variables included in the multivariable logistic regression model were selected a priori based on their clinical relevance, evidence from previous literature, and the objectives of the study. Findings were reported as adjusted odds ratios (AORs) with 95% confidence intervals (CIs), and a two-tailed p < 0.05 defined statistical significance. This manuscript was prepared in accordance with the Strengthening the Reporting of Observational Studies in Epidemiology (STROBE) guidelines for cross-sectional studies [14].

Ethical Considerations

The study received Institutional Ethics Committee approval before commencement. Written informed consent was obtained from participants aged 18 years and above. For participants younger than 18 years, written parental or guardian consent and participant assent were obtained. Participant identity and data were kept strictly confidential, and all identifiers were removed from the analytical dataset.

## Results

Sociodemographic characteristics

The 550 participants had a mean age of 17.93 ± 0.76 years, with a near-equal sex distribution (255 males (46.36%) and 295 females (53.64%)). Participants aged 18 years (n = 230; 41.82%) constituted the largest age group. Most participants identified as Hindu (n = 524; 95.27%), resided off-campus (n = 476, 86.55%), and were equally distributed between professional and non-professional colleges (275 each, 50.00%). Table 1 summarizes the complete sociodemographic profile of the study population, including caste category, parental education and occupation, family type, number of siblings, parental marital status, and socioeconomic status.

**Table 1:** Sociodemographic distribution of the study participants (N = 550) Socioeconomic status was assessed using the Modified Kuppuswamy scale 2024 [10]

Variable	Frequency (n)	Percentage (%)
Age (years)		
17	178	32.36
18	230	41.82
19	142	25.82
Gender		
Male	255	46.36
Female	295	53.64
Religion		
Hindu	524	95.27
Muslim	12	2.18
Christian	10	1.82
Other	4	0.73
Caste category		
General/unreserved	264	48
Other backward classes (OBC)	165	30
Scheduled caste (SC)	99	18
Scheduled tribe (ST)	22	4
Residence		
Hostel	74	13.45
Nonhostel	476	86.55
College type		
Professional	275	50
Nonprofessional	275	50
Family type		
Nuclear	301	54.73
Joint	230	41.82
Extended	19	3.45
Number of siblings		
None	44	8
One	286	52
Two	165	30
Three or more	55	10
Father's education		
Primary school or below	66	12
Middle/high school	143	26
Intermediate/diploma	121	22
Graduate	154	28
Postgraduate or higher	66	12
Father's occupation		
Unemployed/retired	22	4
Agriculture/farming	88	16
Skilled/unskilled worker	99	18
Professional/service	165	30
Business/self-employed	176	32
Mother's occupation		
Homemaker	396	72
Agriculture/labor	33	6
Business/self-employed	33	6
Professional/service	88	16
Parental marital status		
Married	523	95.09
Separated/divorced/widowed	27	4.91
Socioeconomic status		
Upper	18	3.27
Upper middle	85	15.45
Lower middle	195	35.45
Upper lower	192	34.91
Lower	60	10.91

Prevalence and severity of anxiety

**Figure 1:**
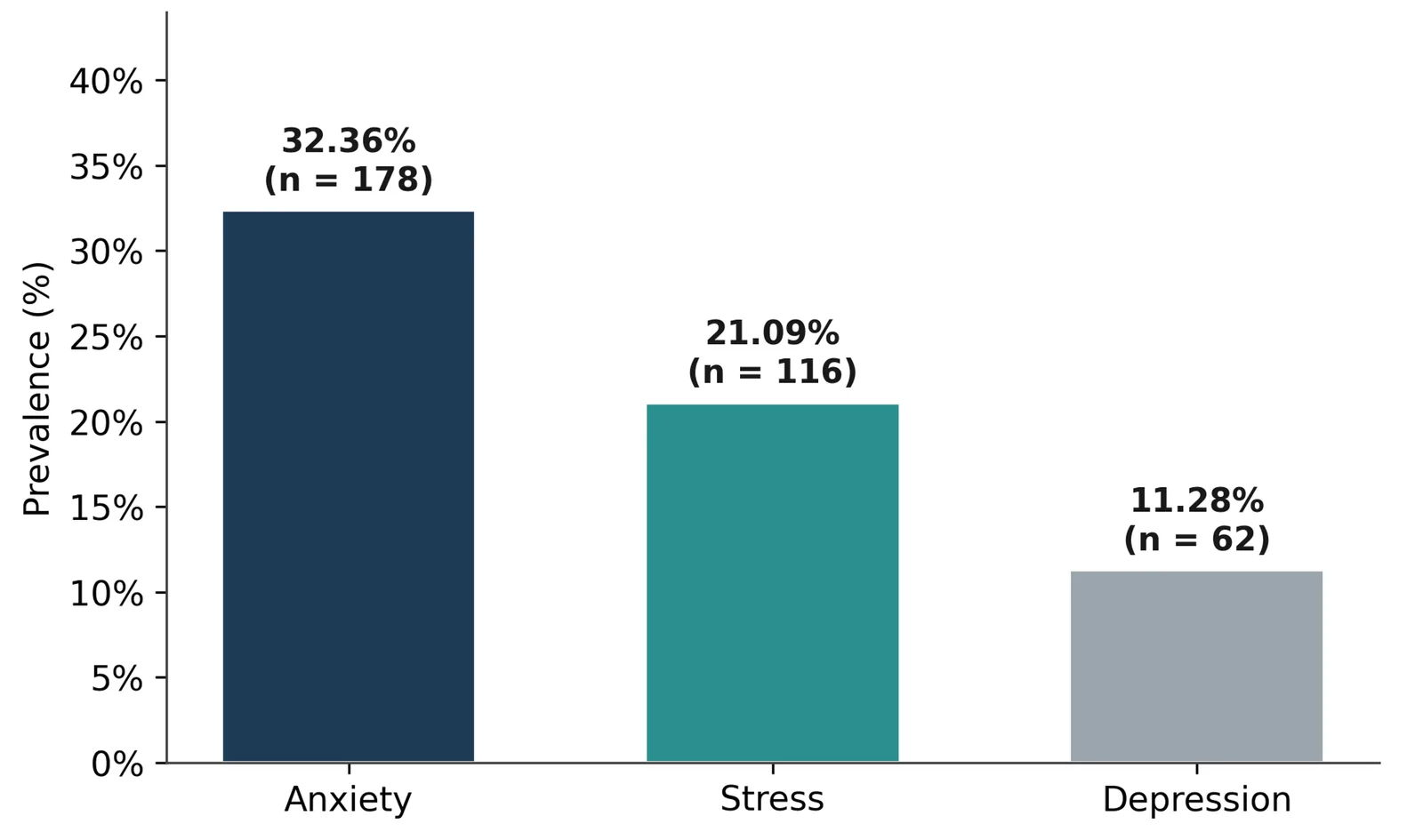
Prevalence of clinically significant anxiety, stress, and depression according to the DASS-21 among study participants (N = 550) DASS-21: Depression Anxiety Stress Scale-21

Of the 550 participants, 372 (67.64%) fell within the normal anxiety range, while 178 (32.36%) met criteria for clinically significant anxiety: mild in 110 (20.00%), moderate in 57 (10.36%), and severe in 11 (2.00%). No participant recorded an extremely severe score. Across the three DASS-21 subscales, anxiety was the most prevalent domain (178; 32.36%), followed by stress (116; 21.09%) and depression (62; 11.28%) (Figure 1, Table 2).

**Table 2:** Distribution of anxiety severity among study participants (N = 550) DASS-21: Depression Anxiety Stress Scale-21 Severity categories were based on DASS-21 anxiety-subscale cutoffs and do not constitute a clinical diagnosis

Anxiety level	DASS-21 score	Frequency (n)	Percentage (%)
Normal	0-3	372	67.64
Mild	4-5	110	20.00
Moderate	6-7	57	10.36
Severe	8-9	11	2.00
Extremely severe	≥10	0	0.00

Family-Related Factors and Anxiety

A statistically significant association was found between family type and anxiety (χ² = 11.406, df = 4, p = 0.0224; Cramér's V = 0.1018). Nuclear-family students showed the highest anxiety prevalence (113 of 301 (37.54%)), compared with 6 of 19 (31.58%) in extended families and 59 of 230 (25.70%) in joint families. Anxiety prevalence varied across sibling-count categories, with 20 of 53 only children (37.74%), 47 of 167 students with one sibling (28.14%), and 53 of 176 students with two siblings (30.11%) meeting criteria for clinically significant anxiety; however, this comparison was not formally tested and is presented descriptively (Table 3).

**Table 3:** Association between family-related variables and clinically significant anxiety (N = 550) V: Cramér's V Percentages represent the proportion within each category with clinically significant anxiety

Variable	Category (n)	Clinically significant anxiety (%)	Association
Family type	Nuclear (301)	37.54	χ² = 11.406; df = 4; p = 0.0224; V = 0.1018
	Joint (230)	25.70	
	Extended (19)	31.58	
Number of siblings	Only child (53)	37.74	Descriptive only (not statistically tested)
	One sibling (167)	28.14	
	Two siblings (176)	30.11	

Anxiety severity also differed significantly across paternal education categories (χ² = 29.507, df = 18, p = 0.0425; Cramér's V = 0.1337). However, some categories had small sample sizes, particularly the postgraduate group (n = 10); therefore, category-specific percentages should be interpreted cautiously (Table 4).

**Table 4:** Association between paternal education and anxiety severity (N = 550) χ² = 29.507; df = 18; p = 0.0425; Cramér's V = 0.1337 Values are presented as n (%); percentages are row-wise proportions within each paternal-education category. No participant had extremely severe anxiety

Father's education	Normal	Mild	Moderate	Severe
Illiterate (n = 120)	81 (67.50)	26 (21.67)	10 (8.33)	3 (2.50)
Primary (n = 65)	42 (64.62)	13 (20.00)	8 (12.31)	2 (3.08)
Middle (n = 123)	81 (65.85)	30 (24.39)	10 (8.13)	2 (1.63)
High school (n = 139)	95 (68.35)	25 (17.99)	18 (12.95)	1 (0.72)
Diploma (n = 16)	7 (43.75)	4 (25.00)	3 (18.75)	2 (12.50)
Graduate (n = 77)	62 (80.52)	9 (11.69)	6 (7.79)	0 (0.00)
Postgraduate (n = 10)	4 (40.00)	3 (30.00)	2 (20.00)	1 (10.00)

Sex and Anxiety

Differences by sex were pronounced and statistically significant (χ² = 15.106; df = 3; p = 0.0017; Cramér's V = 0.1657). Normal anxiety scores were recorded in 192 of 255 males (75.29%) versus 180 of 295 females (61.02%). Mild anxiety was nearly twice as prevalent in females (76 of 295; 25.76%) compared with males (34 of 255; 13.33%), and moderate anxiety was also higher among females (33 of 295; 11.19%) than males (24 of 255; 9.41%) (Table 5).

**Table 5:** Distribution of anxiety severity by sex (N = 550) χ² = 15.106; df = 3; p = 0.0017; Cramér's V = 0.1657

Sex	N	Normal (%)	Mild (%)	Moderate (%)	Severe (%)
Male	255	192 (75.29)	34 (13.33)	24 (9.41)	5 (1.96)
Female	295	180 (61.02)	76 (25.76)	33 (11.19)	6 (2.03)

Factors Independently Associated With Clinically Significant Anxiety

On multivariable binary logistic regression, female sex was independently associated with clinically significant anxiety (AOR: 2.26; 95% CI: 1.45-3.52; p < 0.001), as were professional-college enrolment (AOR: 1.45; 95% CI: 1.05-2.01; p = 0.024) and hostel residence (AOR: 1.68; 95% CI: 1.15-2.45; p = 0.007). Joint-family structure was associated with lower odds of anxiety relative to nuclear families (AOR: 0.62; 95% CI: 0.42-0.91; p = 0.015), whereas the extended-family category did not differ significantly from nuclear families. Within paternal education, diploma-level education was associated with higher odds (AOR: 2.45; 95% CI: 1.10-5.45; p = 0.028) and graduate-level education with lower odds (AOR: 0.55; 95% CI: 0.32-0.95; p = 0.031) relative to illiterate fathers; the postgraduate estimate was imprecise and not statistically significant (AOR: 2.85; 95% CI: 0.85-9.55; p = 0.089). Age and socioeconomic status were not independently associated with anxiety (Table 6).

**Table 6:** Multivariable logistic regression analysis of factors independently associated with clinically significant anxiety (N = 550) AOR: adjusted odds ratio; CI: confidence interval; Ref.: reference category. *statistically significant at p < 0.05 All variables listed were entered simultaneously into the multivariable model; each AOR is adjusted for all other variables in the table

Variable	AOR	95% CI	p-value
Sex			
Male	Ref.	-	-
Female	2.26	1.45-3.52	<0.001*
Age (years)			
17	Ref.	-	-
18	1.12	0.85-1.48	0.415
19	1.34	0.95-1.89	0.092
College type			
Nonprofessional	Ref.	-	-
Professional	1.45	1.05-2.01	0.024*
Residence			
Nonhostel	Ref.	-	-
Hostel	1.68	1.15-2.45	0.007*
Socioeconomic status			
Lower	Ref.	-	-
Upper lower	0.92	0.65-1.30	0.635
Lower middle	0.85	0.58-1.24	0.402
Upper middle	0.72	0.45-1.15	0.170
Upper	0.61	0.28-1.32	0.210
Family type			
Nuclear	Ref.	-	-
Joint	0.62	0.42-0.91	0.015*
Extended	0.81	0.35-1.88	0.620
Paternal education			
Illiterate	Ref.	-	-
Primary	1.10	0.65-1.86	0.721
Middle	1.05	0.71-1.55	0.805
High school	0.95	0.65-1.38	0.785
Diploma	2.45	1.10-5.45	0.028*
Graduate	0.55	0.32-0.95	0.031*
Postgraduate	2.85	0.85-9.55	0.089

Coping Strategies Reported for Anxiety

**Figure 2:**
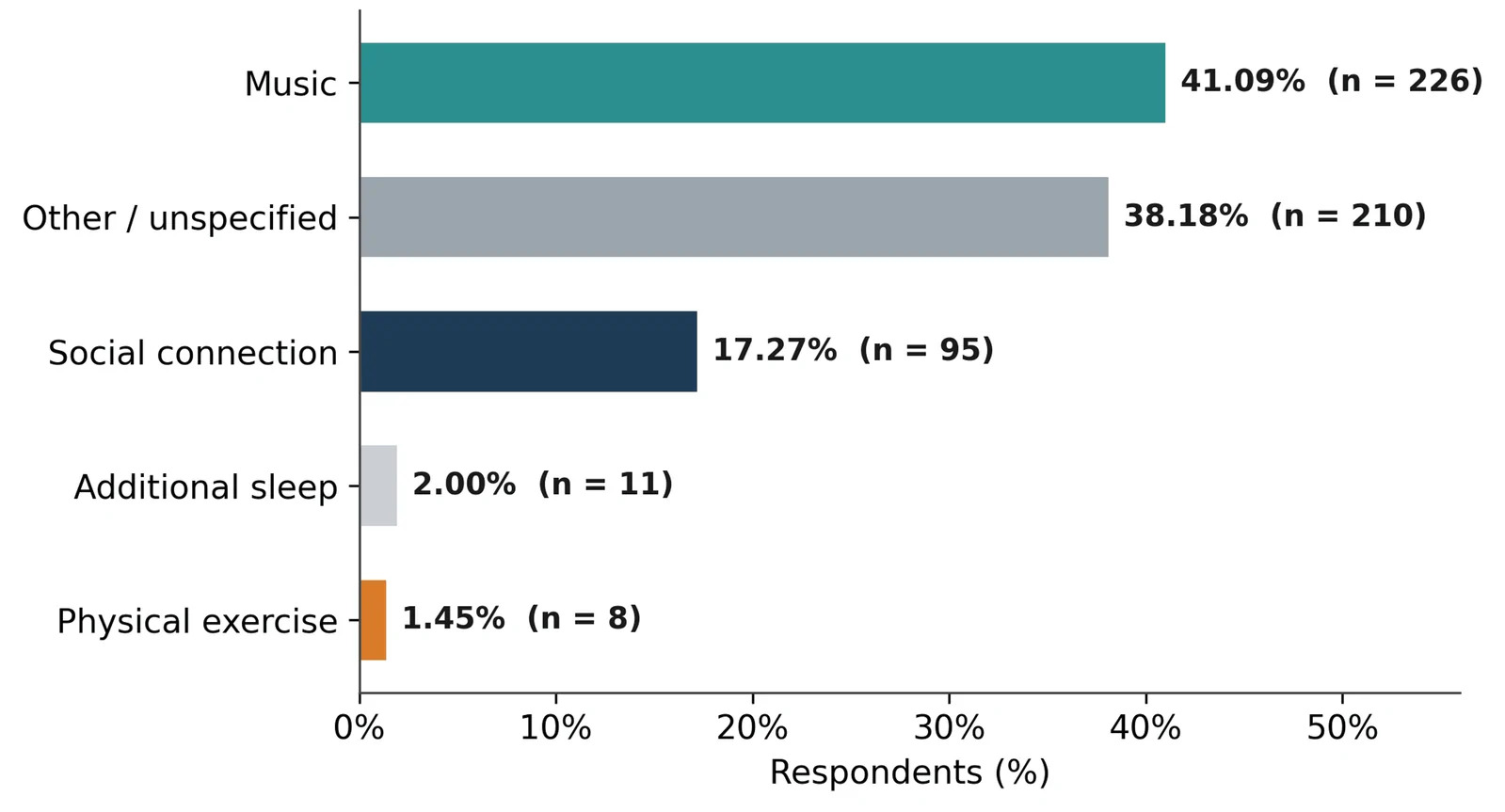
Self-reported coping strategies used by participants to manage anxiety (N = 550) Participants selected the coping strategy they most commonly used

Music was the most frequently reported coping strategy (226; 41.09%), followed by unspecified approaches grouped as "other" (210; 38.18%), connecting with friends or family (95; 17.27%), additional sleep (11; 2.00%), and physical exercise (8; 1.45%) (Figure 2).

## Discussion

The anxiety prevalence of 32.36% observed in the present study is broadly consistent with the findings from Indian undergraduate populations. Iqbal et al. reported anxiety in 66.9% of medical undergraduate students [5]. Similarly, Karmakar et al. reported anxiety among 96.8% of medical students and 58.7% of engineering students in Northeast India [15]. The comparatively lower prevalence observed in the present study may be attributed to differences in the study population, inclusion of both professional and nonprofessional college students, use of the DASS-21 with different scoring thresholds, and variations in academic environments and participant characteristics.

The finding that nuclear-family students carry the greatest anxiety burden (37.54%), and the corresponding lower adjusted odds among joint-family students (AOR: 0.62), is consistent with Indian adolescent data linking family structure and living arrangements to internalizing symptoms [7]. The observed association may reflect differences in social support, household dynamics, or other unmeasured family characteristics. These mechanisms were not directly assessed in the present study. Only children had a higher descriptive prevalence of anxiety than students with one or two siblings. Because this comparison was not formally tested, the findings should be interpreted cautiously. Similar findings have been reported by Cheng et al., who observed that only children had a significantly higher likelihood of experiencing anxiety and depressive symptoms than students with siblings, suggesting that family structure and sibling relationships may influence psychological well-being among college students [16].

Professional-college enrolment was independently associated with higher odds of anxiety (AOR: 1.45). Similar findings were reported by Karmakar et al., who observed a significantly higher prevalence of anxiety among medical students (96.8%) than engineering students (58.7%), suggesting that students in professionally demanding courses experience a greater psychological burden [15]. Likewise, Iqbal et al. reported that 66.9% of medical undergraduates experienced anxiety, with higher anxiety scores among students dissatisfied with their academic performance and those in the earlier semesters of training [5]. Hostel residence was independently associated with higher odds of clinically significant anxiety (AOR: 1.68). However, as the present study did not evaluate factors related to hostel living, the reasons underlying this association remain unclear and warrant further investigation. The association between paternal education and anxiety should be interpreted cautiously because the observed pattern was nonmonotonic, several categories had small cell sizes, and paternal education may reflect broader socioeconomic and family characteristics rather than exerting an independent effect on anxiety [17]. With increasing reliance on digital learning resources among health-profession students, academic demands may also be shaped by screen-based learning, limited interaction with teachers, and connectivity-related barriers; however, the relationship between these factors and anxiety requires dedicated evaluation [18].

The disparity in anxiety by sex is among the most consistent findings in psychiatric epidemiology. The roughly 2.26-fold higher adjusted odds in females (p < 0.001) are consistent with Indian undergraduate data, in which female sex is repeatedly associated with higher anxiety scores [5], and are consistent with global epidemiological studies reporting a higher prevalence of anxiety disorders among females [19]. The higher prevalence of anxiety among female students observed in the present study is consistent with previous findings among Indian adolescents. Singh et al. reported higher anxiety levels among females and suggested that demographic and psychosocial factors, including family environment and self-concept, may contribute to these differences [7]. Similar sex-related differences in anxiety have also been reported in epidemiological studies, although the present study did not investigate the underlying mechanisms [19].

The dominance of music as a coping strategy (41.09%) is explicable by its accessibility, cultural familiarity, and immediate capacity to modulate mood, consistent with evidence that young people commonly use music listening to self-manage anxiety [20]. The use of social connection (17.27%) aligns with Indian community data showing that close family and community ties are associated with lower odds of anxiety and depression [21]. Meta-analytic evidence suggests that resistance exercise can reduce anxiety symptoms [22]. The reasons for the low reporting of exercise as a coping strategy were not assessed and should be explored in future studies.

Limitations

This study has several limitations. Its cross-sectional design precludes causal inference. Academic stress and family environment were captured through proxy sociodemographic indicators rather than validated stress or family-functioning scales, which may incompletely represent these constructs. Reliance on self-report introduces potential recall and social desirability bias, and the single-district sampling frame may limit generalizability beyond comparable central Indian settings. The sibling-count gradient in anxiety prevalence was descriptive and not subjected to formal statistical testing and should be interpreted with corresponding caution. The DASS-21 identifies symptom severity rather than clinical diagnoses of anxiety disorders. Students were sampled from 10 colleges, but clustering at the institutional level was not accounted for during regression modelling. Consequently, standard errors may have been underestimated, and CIs may therefore be slightly narrower than the true values.

Strengths of the study

The present study has several strengths. It included a relatively large sample of first-year college students drawn from both professional and nonprofessional colleges using a multistage sampling strategy, thereby improving the representativeness of the study population. Anxiety was assessed using the validated DASS-21 instrument, which is a widely accepted tool for measuring anxiety symptoms in adolescents and young adults. Furthermore, the study examined a broad range of sociodemographic, family-related, and academic factors, allowing identification of independent predictors of clinically significant anxiety through multivariable logistic regression. These findings contribute valuable evidence on the burden and correlates of anxiety among college-going adolescents in an understudied district of central India and may help inform institution-based mental health screening and intervention strategies.

Implications, recommendations, and future directions

The findings of this study have important implications for college health services and public health practice. The high prevalence of anxiety among first-year college students highlights the need for routine mental health screening, particularly during the transition to college. Institutions should consider implementing regular screening programs, accessible counselling services, stress management workshops, and peer-support initiatives, with particular attention to students enrolled in professional courses and those residing in hostels. Faculty members should also be sensitized to recognize early signs of psychological distress and facilitate timely referral for appropriate care. Future research should employ longitudinal study designs to establish temporal relationships between potential risk factors and anxiety, include a wider range of colleges across different geographic regions to enhance generalizability, and investigate additional determinants such as academic performance, social support, lifestyle behaviors, coping strategies, and digital media use. Intervention studies evaluating the effectiveness of institution-based mental health promotion programs are also warranted.

## Conclusions

Anxiety symptoms were common among college-going adolescents in Ratlam district, with clinically significant anxiety reported by 32.36% of participants. Female sex, professional-college enrolment, hostel residence, family type, and paternal education were associated with anxiety in this sample. Because the study was cross-sectional and academic- and family-related constructs were assessed using proxy variables, causal inferences cannot be made. The findings support strengthening campus-based mental health screening, expanding access to counselling services, and promoting healthy coping strategies. Longitudinal studies using validated measures of academic stress and family functioning are needed.
